# Psychological Discomfort in Nursing Degree Students as a Consequence of the COVID-19 Pandemic

**DOI:** 10.3390/jcm10235467

**Published:** 2021-11-23

**Authors:** Fernando Espina-López, Emilia Moreno-Sánchez, Francisco-Javier Gago-Valiente, Jesús Sáez-Padilla, Vanesa Salado-Navarro, María-de-los-Ángeles Merino-Godoy

**Affiliations:** 1 Huelva-Costa Primary Care District, 21007 Huelva, Spain; fernandoeslo99@gmail.com; 2 Department of Pedagogy, Faculty of Education, Psychology and Sports Sciences, University of Huelva, 21007 Huelva, Spain; emilia@dedu.uhu.es; 3 Preventive Medicine and Public Health Area, University of Huelva, 21071 Huelva, Spain; 4 Integrated Didactics Department, Faculty of Education, Psychology and Sports Sciences, University of Huelva, 21071 Huelva, Spain; jesus.saez@dempc.uhu.es; 5 Department of Experimental Psychology, Faculty of Psychology, University of Seville, 41004 Seville, Spain; vsalado@us.es; 6 Nursing Department, Faculty of Nursing, University of Huelva, 21007 Huelva, Spain; angeles.merino@denf.uhu.es

**Keywords:** COVID-19, pandemic, nursing, students, stress, anxiety, depression, psychological discomfort

## Abstract

Students are a population at risk of developing psychological complications, such as psychological discomfort, stress, and anxiety, among other problems, especially during the current health crisis due to the COVID-19 pandemic. The present study’s objective was to analyze the effects of the COVID-19 pandemic on the psychological discomfort of final-year nursing students. A cross-sectional descriptive observational study was carried out. To analyze the psychological discomfort of the participants, the Kessler test (previously validated) was used. The results of this test were divided into two levels (High ≥ 21/Low < 21), showing high sensitivity as a screening method for anxiety and depression. Questionnaires were sent via email to final-year nursing students of Spanish and South American universities, inviting them to participate voluntarily. The sample consisted of 400 students, with an average age of 23.29 years and a sex proportion of 82.75% women and 17.28% men. Almost all participants (*n* = 396) belonged to Spanish universities, and the greatest participation corresponded to Andalusian universities (64.5%). The average psychological discomfort was high (M = 27.94). Statistically significant relationships were detected between age, sex, and feeling ready for the world of work, observing no relationships with the rest of the studied variables. The sample of 4th-year students of the Degree of Nursing presented a high level of psychological discomfort. This pathology does not seem to be related to having suffered from COVID-19 or being in contact with infected people during the practicum and is more strongly related to personal sociodemographic variables and students’ preparation for the world of work.

## 1. Introduction

Since the 1918 Spanish Influenza pandemic, which caused approximately 40 million deaths [1], no other pandemic has impacted society as much as the SARS-CoV-2 virus. Its repercussions have demonstrated the frailty of every vital scope. The rapid spread of the new SARS-CoV-2 corona virus has forced the adoption of exceptional prevention and containment measures.

In Spain, after the declaration of the World Health Organisation (WHO) recognizing the pandemic due to the disease caused by the SARS-CoV-2 coronavirus (COVID-19) on 11 March 2020, one of the greatest challenges of recent decades was confronted [2]. To face this world crisis, social, economic, and political resources were demanded, as well as the recognition of the pandemic’s possible consequences, requiring an approach to contain the outbreak through basic principles of public health [3].

It is important to highlight that, although the reaction was proportional to different crisis levels [4], difficult situations took place in hospitals due to the high contagion rates. In Spain, the coronavirus expanded from February 2020, and, at the middle of March 2020, an emergency state was imposed, with the aim of containing the contagions and reduce the overload of patients that occurred in hospitals. For over two months, the population went through a strict confinement, which did not prevent the health services from being overwhelmed in many places before flattening the curve of contagion [4]. From the beginning of the pandemic, the virus spread at a great rate throughout the entire territory; however, it did not affect all regions equally. This geographical “inequality” is not only obvious in the vaccination rate or in the parameter of cumulative disease incidence but also in the mortality rate [5].

It is known and proven that the COVID-19 pandemic has caused certain changes in the world population that challenge all aspects of “usual life”. Daily actions from before this historical event have changed, and such change has impacted society in terms of personal affairs, human relationships, employment, education, connections with the community, and other social activities [6].

Many countries implemented several isolation measures to prevent the spread of the outbreak until a vaccine was available or until the percentage of vaccinated population increased, which led to important social and economic consequences in the entire community, resulting in negative psychological repercussions. These measures include home isolation, social distancing, closing of educational centers, universities, and businesses, cancellation of events, conferences, and seminars, postponement of sports events, and travel restrictions [3,6].

In fact, the spread of the outbreak of the new 2019 coronavirus SARS-CoV-2 brought a drastic change to all humanity, with a considerable psychosocial impact on the professions related to health and on the university students of health sciences [7].The impact on health professionals has been reported in different Spanish and international studies [8,9,10]. Roberts, Mc Aloney-Kocaman, Lippiett, Ray, Welch, and Kelly [11] state that there were higher levels of anxiety and depression among nurses with less experience.

It is also important to point out the impact of the state of health emergencyon educational contexts at all stages.

The current pandemic has caused vast and prolonged interruptions in the teaching–learning processes, in the normal support systems, and in the social activities of students, with ongoing consequences that reflect the distance fromrecovering the past normality in the near future [6].

This impact on students’ lives is due to the convergence of many variables related to the interruption of students’ university life and other aspects of their everyday life, including family and social life and their capacity to participate in habitual activities such as sports [6].

University students can be one of the most affected populations. Students have modified their routines due to the COVID-19 pandemic [12]. For many students, such as those of the Degree of Nursing, the requirements of isolation and social distancing have caused the cancellation of face-to-face lectures and exams, practicums in healthcare centers, and part-time jobs [6].

Attending lectures and other activities related to studying areof vital importance for professional training. For many students, going to university is a way of learning to face reality, as well as a way of maintaining their relationships with peers, friends, and faculty members [13].

Some students prefer to attend face-to-face lectures over the online modality, due to their personal learning styles, the capacity to learn “face-to-face” to increase their learning confidence, or having access to the support from their peers. For those who do not study in their locality of residence, in a shared flat/house, or in student accommodations, not attending face-to-face lectures increases their need for economic investment in technological devices to be able to attend the online lectures. Moreover, if they could not attend the face-to-face lectures, students suffered the subsequent loneliness and loss of connection with other people related to their studies (students and faculty members) [13].

In some countries, nursing students experienced situations under certain pressure, where they had to assume the role of health professionals, assisting upon request in the units where they were assigned, with critical patients, putting students’ mental health at risk due to these stressful situations [14]. In fact, it has been asserted that the COVID-19 pandemic has placed a considerable number of nursing students in a situation in which they feel that they must choose between safety outside of the healthcare profession or continuing their nursing studies [15]. This dilemma can cause additional psychological distress among nursing students and, as in the case of previous global disease outbreaks such as Ebola, healthcare professionals are at great risk of developing burnout, fatigue, lower job satisfaction and morale, and working stress during pandemics [6].

Therefore, it has been demonstrated that nursing students experience greater anxiety than students of other degrees, due to the specific characteristics of their training [16]. This may be caused by the additional stress factors related to the social and academic adjustments derived from the COVID-19 pandemic within the educational community [17,18].

Several studies on this population report that those aged between 18 and 20 years, mostly women, show higher stress levels. It was found that watching the news about the pandemic, concern about infection, and the time restrictions imposed by the curfew influenced nursing students’ stress levels [3].

It is known that the transition of nursing students to the role of healthcare professionals has always been stressful, thus, there are people who have abandoned their profession when it was time for them to practice it. Currently, the COVID-19 pandemic has posed a new and serious stress and anxiety factor to nursing students. These students have lived through situations such as the struggle of their peers who work in healthcare centers with the isolation measures. For example, one of these situations was the lack of basic material, including personal protection equipment (PPE), along with the direct exposure to COVID-19 patients. This may have caused negative perceptions toward nursing students’ future occupation [19].

Therefore, several actions have been proposed to implement a solid and suitable support plan aimed at preventing professional discomfort [14]. In this preventive line, it has been stated that study plans should include specific content on the management of pandemics and other disasters, in order to increase the preparation of the students, i.e., the future healthcare professionals, for these types of scenarios [20].

Hernández-Martínez, Rodríguez-Almagro, Martínez-Alce, Romero-Blanco, García-Iglesias, and Gómez-Salgado [2] claim that, although nursing students showed willingness to incorporate into the healthcare centers, they felt unprepared to work in the field of intensive care and demanded further training to improve their levels of anxiety and stress regarding the care of critical patients. Guidance, followup, and emotional support in critical situations have proved key to overcome stressful situations [21].

For all these reasons, the aim of the present study was to analyze the influence of the COVID-19 pandemic on the psychological discomfort of final-year students of the Degree of Nursing due to the temporal proximity of their professional practice. The study’sobjective was to determine the prevalence of psychological discomfort in the study population, as well as the influence of sociodemographic and academic variables. The pandemic has had a direct impact on this new generation of nursing professionals. Discovering the possible psychological repercussions in these students will enable implementation of psychological and didactic corrective measures that reduce the negative effects on their training and personal health. Such measures could facilitate the integration of those who are about to become healthcare professionals, thus contributing to improving the quality of healthcare services.

## 2. Materials and Methods

### 2.1. Participants

This was a descriptive cross-sectional study with 400 nursing students, of whom 82.75% were women (*n* = 331) and 17.25% were men (*n* = 69). It is important to take into account that most nursing students are females; therefore, in the proportion of samples in studies related to these professionals, the representation of women is considerably greater than that of men. The average age of the participants was 23.29 years. Most of the participants (*n* = 396) belonged to Spanish universities, and the greatest participation was from Andalusian universities (64.5%) (students from national and international universities that taught the degree of nursing participated in the study; the origin and number of participants is shown in Annex II). Regarding the sociodemographic characteristics, it is important to highlight that 60% of the students lived with their families (*n* = 232), 30.75% lived with other university students (*n* = 123), and 8.75% lived alone (*n* = 35).

### 2.2. Procedure

All students selected were in the fourth year of the degree of nursing. The study excluded those who did not perform the practicum in healthcare centers, since they did not experience the same situations.

To calculate the sample size, we determined the minimum sample required to estimate a parameter with a proportion of 0.50 (scenario with the greatest sample requirement) in a population of unknown size. Taking these data into account, and considering a 95% confidence level, 10% precision, and 5% of possible losses during the process of data cleansing, we calculated that the minimum sample size for this study would be 120 individuals. However, to carry out more complex analyses that involve the segmentation of the sample into subgroups of individuals, we decided to obtain a sample of around 400 individuals.

The data were gathered through self-administered questionnaires. The fieldwork began on 10 March 2021 and was terminated on 23 April 2021. The questionnaire was sent in a crucial moment of the pandemic, where the number of cases increased constantly and severe restrictions were established.

Student representatives of universities of different European and South American countries were contacted through social networks and via email and telephone. Representatives distributed the questionnaires among the final-year students of the degree of nursing who agreed to participate voluntarily. The format of the instrument was developed using Google Forms and the data were gathered directly from said platform.

Written informed consent was obtained from all participants for the publication and analysis of the data. Participant anonymity was guaranteed, thus complying with the current regulations on user data protection, specified in Organic Law 3/2018, of 5 December, on the Protection of Personal Data and the Guarantee of Digital Rights. Moreover, the formal aspects of the declaration of Helsinki were considered at all times. The study was approved by the Research Ethics Committee of Huelva (code: MG-COV-2021-02; internal code: 0742-N-21).

### 2.3. Evaluation Instruments

The participants completed a brief sociodemographic questionnaire, through which they provided information about their age and sex, the university in which they studied, and their place of residence during the academic year. The questionnaires were distributed through Gmail, thus participants had to provide their email address, which prevented duplicates. Additional items were introduced to gather information about the training of the participants, whether they had performed the practicum, whether they felt ready for the world of work, and whether they had suffered from the disease caused by SARS-CoV-2 (COVID-19).

The main variable of this present study was psychological discomfort, and it was measured using the Kessler scale (K-10) [22].This scale shows the level of psychological discomfort of the users during the 4-week period before completing the scale. Psychological discomfort is defined as the level of stress, demoralization, malaise, and unrest perceived in oneself [23].

Since the Kessler scale (K-10) is also used as a screening method for depression and anxiety, these two variables were also measured. Anxiety is defined as a human emotion present in most mental and medical disorders that appears as a response to the perception of a threat or danger. It helps people to prepare and practice in order to improve their activity and thus adopt the appropriate caution measures against potentially dangerous situations. Clinically, anxiety is fear without a known cause [24]. On the other hand, depression is understood as a mood disorder that causes symptoms of distress and affects how the person feels, thinks, and coordinates the activities of daily living, such as sleeping, eating, and working. To be diagnosed with depression, symptoms must be present during most of the day, almost every day, for at least two weeks.

This K-10 scale was selected for its adequacy and adaptation to the study scope. It is a brief questionnaire that can be easily applied by first-level healthcare professionals. K-10 has been translated into Spanish and applied in Spain, Colombia, Mexico, and Peru, as well as in other American and European countries [22].

The Kessler instrument consists of 10 items, with 5 Likert response options each, ordered hierarchically from 1 to 5: “never, almost never, sometimes, almost always and always”. The sum of the scores can range from a minimum of 10 points to a maximum of 50. The interpretation of the scores corresponds to 4 levels as follows: 10–15 points, low level; 16–21, moderate level; 22–29, high level; and 30–50, very high level [22].

K-10 has been previously validated, with high sensitivity and specificity [22]. According to the validation study, the score of the scale can be divided into 2 levels, due to its high specificity and sensitivity, for the subsequent screening for anxiety and depression. This screening also categorizes the participants based on their psychological discomfort, although with two reference levels of anxiety and depression. The cutoff points would be, on the one hand, scores of 10–20 for low affectation of psychological discomfort (anxiety and depression) and, on the other hand, scores of 21–50 for high affectation.

Aranguren and Brenlla [25] explored the discriminant validity of K–10 in psychiatric patients, also comparing the scores obtained in psychiatric patients and non-psychiatric individuals. The results showedadequate validity and reliability of the instrument, with a Cronbach’s alpha of 0.91 for the patients and 0.80 for the control individuals.

### 2.4. Statistical Analysis

The statistical analyses were conducted using IBM SPSS Statistics v.27.0 software. The descriptive analysis was performed with central tendency measures (mean and standard deviation), frequencies and percentages, using frequency tables.

Specifically, for the variables of age, psychological discomfort, anxiety, and depression, the mean and standard deviation were calculated.

The frequencies and percentages were calculated for the variables sex, practicum, university of origin, training, residence during the academic year, having suffered from COVID-19, psychological discomfort, anxiety, and depression.

To study the possible relationship between psychological discomfort and the rest of the variables, after verifying the normality of the quantitative variables, the following tests were carried out:

− Pearson’s Chi-squared, to explore the correlation between psychological discomfort and the variables of COVID-19, sex, coexistence (residence during the academic year), and feeling ready for the world of work (training).
− Student’s t, to analyze the relationship between psychological discomfort (anxiety and depression) and age.
− ANOVA, to study the correlation between psychological discomfort and age.

## 3. Results

### 3.1. Prevalence of Having Suffered from COVID-19 among the Participants

**Figure 1:**
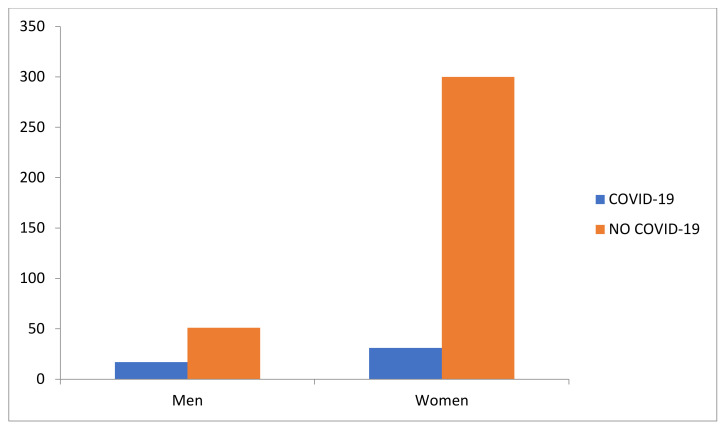
Frequencies of having suffered from COVID-19 based on sex.

A total of 15.25% (*n* = 61) of the participants had suffered from COVID-19 before the study period, whereas 84.75% (*n* = 338) had not. Figure 1 shows these data categorized based on sex.

### 3.2. Association of Psychological Discomfort with Sex, Residence during the Academic Year, Having Suffered from COVID-19, and Feeling Ready for the World of Work

**Figure 2:**
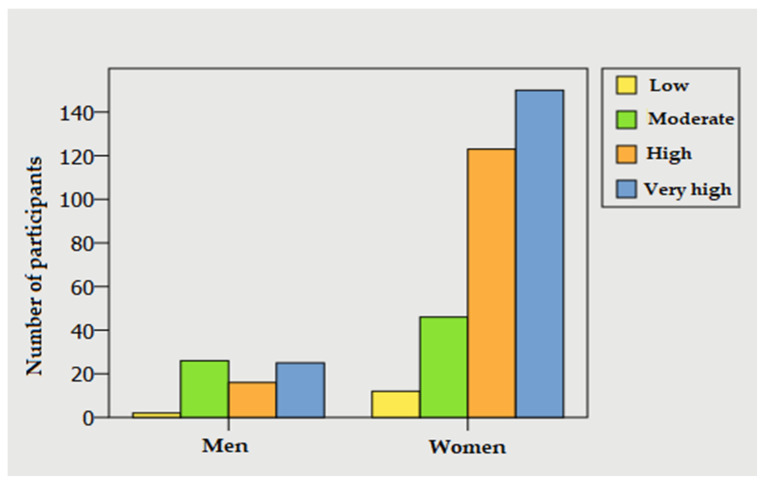
Frequencies in K-10 based on sex.

A Chi-squared test of independency was conducted to determine whether the distribution of psychological discomfort was similar in women and men. The results show significant differences between men and women(X^2^ = 14.958; *p* = 0.002, 95% CI). There was a greater proportion of women with high scores in psychological discomfort (41.7%) compared to men (24.6%) (Figure 2).

Regarding the study of the correlation between psychological discomfort and residence during the academic year, it was observed that the two variables were correlated (X^2^ = 458.823; *p* = 0.026, 95% CI). Table 1 shows the percentages of greatest and least affectation in K-10 (very high level and low level, respectively) as a function of the coexistence unit during the academic year.

In the analysis of the relationship between psychological discomfort and having suffered from COVID-19, no correlation was observed between these variables (X^2^ = 5.652; *p* = 0.130, 95% CI).

**Figure 3:**
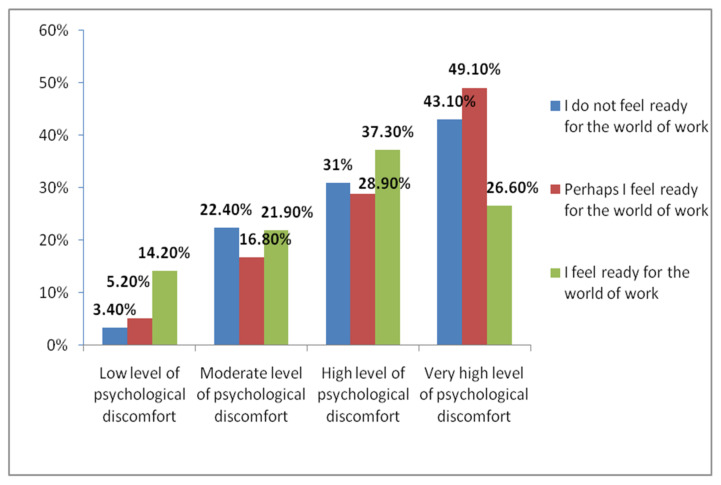
Correlation between psychological discomfort and feeling ready for the world of work.

Lastly, psychological discomfort was correlated with feeling ready for the world of work (X^2^ = 24.873; *p* = 0.000, 95% CI). The participants who responded that “perhaps” they felt ready for the world of work showed greater representation in percentages of very high levels of psychological discomfort. However, the percentages of greatest representation in low levels of psychological discomfort were observed in those participants who responded “yes” to this item (Figure 3).

### 3.3. Correlation between Psychological Discomfort and Age

The results of the single-factor ANOVA between groups showed that the differences in the mean scores of psychological discomfort according to age were not statistically significant (F = 0.712; *p* = 0.545, 95% CI).

### 3.4. Prevalence of Anxiety and Depression (Psychological Discomfort with the 2-Level K-10)

For the screening of anxiety and depression, the score of the scale was divided into two levels [22]: low (10–20 points) and high (21–50 points). Most of the participants presented a high level in the prevalence of anxiety and depression (Table 2).

### 3.5. Correlation between Psychological Discomfort (2-Level K-10) and Age

A Student’s t-test was performed to explore the existence of differences in terms of psychological discomfort (anxiety and depression) according to age. The results showed that there were no statistically significant differences (t (398) = 1.440; *p* = 0.151, 95% CI).

### 3.6. Association of Psychological Discomfort (2-Level K-10) with Sex, Residence during the Academic Year, Having Suffered from COVID-19 and Feeling Ready for the World of Work

A correlation was observed between psychological discomfort and sex (X^2^ = 9.259; *p* = 0.002, 95% CI), with a greater proportion of women (78.2%) who showed a high level of psychological discomfort compared to men (60.9%).

With respect to the analysis of the association of psychological discomfort with residence during the academic year and having suffered from COVID-19, no correlation was observed between these variables (X^2^ = 142.523; *p* = 0.291, 95% CI).

Lastly, a relationship was detected between psychological discomfort and feeling ready for the world of work (X^2^ = 6.905; *p* = 0.032, 95% CI). The participants who claimed to feel ready for the world of work presented a lower level of psychological discomfort (68.6%) compared to those who did not feel ready (81%). The rest of the students who responded “perhaps” in this item represented a percentage of 79.8% in high affectation of anxiety and depression.

## 4. Discussion

The main objective of this study was to analyze the level of psychological discomfort of the final-year nursing students. The results showed that these students obtained high scores in this variable, following the line of research such as Herrera and Rivera [23]. However, other studies have reported a moderate prevalence [26].

The data of this study indicate that those who felt ready and qualified for the world of work presented a lower level of psychological discomfort. These data are consistent with the results of other studies [23], where the mean marks and satisfaction with the degree were also correlated with a low psychological discomfort.

A correlation was observed between sociodemographic variables and psychological discomfort. Moxham, Fernández, Kim, Lapkin, Ten Ham-Baloyi and Mutair [27] stated that the variables related to psychological discomfort are sex, age, marital status, and employment status. Some variables such as coexistence, high marks, and the practicum are not statistically significantly correlated with psychological discomfort [27].

Furthermore, the results of the present study indicate that psychological discomfort affects women to a greater extent than men, which is in line with the findings of Liébana-Presa, Fernández-Martínez, Gándara, Muñoz-Villanueva, Vázquez-Casares and Rodríguez-Borrego [28], who reported that women obtained a higher score than men. It is important to highlight that the data of the present study corroborate the need to considering gender as a key for a change of paradigm that will allow generating more accurate and inclusive scientific knowledge, as well as a more realistic, fair, and egalitarian healthcare system.

As was commented above, in this study, those participants who felt ready for the world of work presented a lower psychological discomfort, which could be related to a greater resilience and a greater capacity to adapt to changes [29].

Having performed the practicum in healthcare centers and having suffered from COVID-19 were not significantly related to psychological discomfort. The data of this study indicate that psychological discomfort is related to sociodemographic variables such as sex, age, and university of origin, as well as preparation for the world of work.

The results of this investigation show the convenience of developing mitigation strategies in pandemic situations. Since resources can be particularly scarce during these states, adequate psychological support could be provided in many different forms, including telemedicine and informal support groups [30].

### Limitations of the Study

This study provides very novel information about a series of indicators of mental health in a student population that had been poorly studied to date in a pandemic situation, thereby updating the knowledge on this topic to contribute to future research lines that address this problem. However, this study presents some limitations that must be pointed out. One of such limitations is that, despite the large sample size, most of the population who responded to the questionnaire were Spanish, thus the data cannot be extrapolated to the international scope. Similarly, participation in this study was voluntary, which can pose a sample selection bias.

Further research in this line is required, with larger populations and greater representation of international origin, in order to extrapolate the results. Moreover, randomized sampling must be applied to avoid selection bias.

## 5. Conclusions

In sum, the final-year nursing students who participated in this study suffered negative psychological consequences during the pandemic. These participants showed a high level of psychological discomfort. The differences in the latter were related to sociodemographic variables such as age, sex, university of origin, and feeling ready for the world of work.

The COVID-19 pandemic has caused serious repercussions. Therefore, in these periods of health crisis, it is necessary to develop research lines focused on studying the phenomenon and its impact with other variables, in order to know its effect on future healthcare professionals. This could help to prevent and address the negative personal aspects that can affect students’ professional integration.

## Figures and Tables

**Table 1:** Percentages of affectation in coexistence units in low and high levels of psychological discomfort.

Coexistence Unit During the Academic Year	Low Level of Psychological Discomfort	Very High Level of Psychological Discomfort
-Two or more roommates		
-Partner and child		
-Father and mother		
-Partner and partner’s child		
-Roommates and partner		
-Father, mother, and sister		
-Parents and siblings		
-Brother		
-Mother and brother		
-Mother, stepfather, and three siblings		
-Friends (females)		
-One or more non-university roommates	0%	100%
-Classmates		
-Mother and siblings		
-Father		
-Student accommodation		
-Maternal grandparents, mother, stepfather, and younger sister		
-Roommates from the same university		
-Daughter		
-Sister	100%	0%
-Mother and sister		
-Father, mother, and brother		

**Table 2:** Frequencies and percentages of the participants in the 2-level K-10.

Range OF K-10 (2 Levels)			
Low		High	
N	%	N	%
63	15.75%	337	84.25%
